# Supplementary material for: Leucocyte-Rich Platelet-Rich Plasma Enhances Fibroblast and Extracellular Matrix Activity: Implications in Wound Healing
Source: Int J Mol Sci. 2020 Sep 6;21(18):6519. doi: 10.3390/ijms21186519 (PMC7556022; doi:10.3390/ijms21186519)
Supplement: Supplementary file 1 [file ijms-21-06519-s001.zip › Supplementary Materials /Table S3.docx]

**Table S3:** LR-PRP Donor Exclusion Criteria List

| Heart disease | Haemodynamic Instability |
| --- | --- |
| High blood pressure | Hypofibrinogenaemia |
| Diabetes | Sepsis |
| Respiratory problems | Acute and Chronic Infections |
| Bowel problems | Chronic Liver Pathology |
| Epilepsy | Anti-Coagulation Therapy |
| Allergies or anaphylactic reactions | Skin Disease or Cancer |
| Platelet Dysfunction Syndrome | Severe Metabolic |
| Critical Thrombocytopenia | Systemic Disorders |
| Taking steroids or aspirin | Drug dependency/alcohol abuse/smoking |
| Taking any other kind of medication | Pregnant or breastfeeding |
| HIV | Hepatitis |
