# Supplementary material for: Leucocyte-Rich Platelet-Rich Plasma Enhances Fibroblast and Extracellular Matrix Activity: Implications in Wound Healing
Source: Int J Mol Sci. 2020 Sep 6;21(18):6519. doi: 10.3390/ijms21186519 (PMC7556022; doi:10.3390/ijms21186519)
Supplement: Supplementary file 1 [file ijms-21-06519-s001.zip › Supplementary Materials /Table S2.docx]

**Table S2.** The formulae used for calculation of PRP characteristics

| **Parameter =** | **Calculation** |
| --- | --- |
| Platelet yield % | (Volume of PRP (ml) x Platelet concentration in PRP) ÷  (Net volume of whole blood collected (ml) x platelet concentration in whole blood) |
| Relative composition  in platelets % | (Platelet concentration in PRP) ÷  (Platelet concentration in PRP + WBC concentration in PRP + RBC concentration in PRP) |
| Relative composition  in WBC % | (WBC concentration in PRP) ÷  (Platelet concentration in PRP + WBC concentration in PRP + RBC concentration in PRP) |
| Relative composition  in RBC % | (RBCs concentration in PRP) ÷  (Platelet concentration in PRP + WBC concentration in PRP + RBC concentration in PRP) |
| Factor increase  in platelets or WBC | Platelet concentration or WBC concentration in PRP ÷  Platelet concentration or WBC concentration in whole blood |
| Platelet dose in PRP | Volume of PRP (ml) x Platelet concentration in PRP |

Abbreviations: Platelet-rich plasma (PRP), white blood cells (WBC), red blood cells (RBC).
