# Supplementary material for: Leucocyte-Rich Platelet-Rich Plasma Enhances Fibroblast and Extracellular Matrix Activity: Implications in Wound Healing
Source: Int J Mol Sci. 2020 Sep 6;21(18):6519. doi: 10.3390/ijms21186519 (PMC7556022; doi:10.3390/ijms21186519)
Supplement: Supplementary file 1 [file ijms-21-06519-s001.zip › Supplementary Materials /Table S1.docx]

**Table S1:** Haematological values: Whole blood, LR-PRP and LP-PPP.

| **Cell counts in mean ± SEM** | **Whole Blood** | **LR-PRP** | **LP-PPP** |
| --- | --- | --- | --- |
| Platelet x 10^3^/µl | 167.20±14.39 | 1422.00±317.21 | 55.33±10.13 |
| WBC x 10^3^/µl | 4.52±0.43 | 16.36±2.08 | 0.08±0.02 |
| Monocyte x 10^3^/µl | 0.37±0.03 | 2.38±0.25 | Negligible |
| Lymphocyte x 10^3^/µl | 1.27±0.14 | 7.05±0.28 | Negligible |
| Neutrophil x 10^3^/µl | 2.62±0.47 | 1.40±0.32 | 0.03±0.02 |
| RBC x 10^6^/µl | 4.36±0.48 | 0.35±0.06 | 0.003±0.002 |
| Haematocrit (%) | 38.93±3.66 | 3.06±0.54 | 0.03±0.02 |
| Platelet dose in LR- PRP | NA | 9.95x10^9^  | 3.85x^3^ |
| **Characteristics in mean ± SEM %** | | **LR-PRP %** | **LP-PPP %** |
| Platelet yield | | 102.10±22.36 | 4.60±1.22 |
| WBC yield | | 40.75±5.33 | Negligible |
| Monocyte yield | | 77.57±9.16 | Negligible |
| Lymphocyte yield | | 68.11±6.41 | Negligible |
| Neutrophil yield | | 6.21±1.16 | Negligible |
| Factor increase in platelet concentration | | 8.75±1.92 | Negligible |
| Factor increase in WBC concentration | | 3.48±0.45 | Negligible |
| Relative composition in platelets | | 98.76±0.17 | 1.00±0 |
| Relative composition in WBC | | 1.19±0.16 | Negligible |
| Relative composition in RBC | | 0.03±0.01 | Negligible |
| Platelet dose in PRP | | 9.95x10^9^  | 3.85x10^3^  |

Abbreviations: Leucocyte-rich, platelet-rich plasma (LR-PRP), leucocyte-poor, platelet poor plasma (LP-PPP), white blood cells (WBC), red blood cells (RBC), SEM, standard error of the mean.
