# Supplementary material for: Leucocyte-Rich Platelet-Rich Plasma Enhances Fibroblast and Extracellular Matrix Activity: Implications in Wound Healing
Source: Int J Mol Sci. 2020 Sep 6;21(18):6519. doi: 10.3390/ijms21186519 (PMC7556022; doi:10.3390/ijms21186519)
Supplement: Supplementary file 1 [file ijms-21-06519-s001.zip › Supplementary Materials /Figure S1.docx]

**Figure S1**- Expressions of mRNA genes of human ECM and adhesion molecules

LR-PRP versus LP-PPP

| **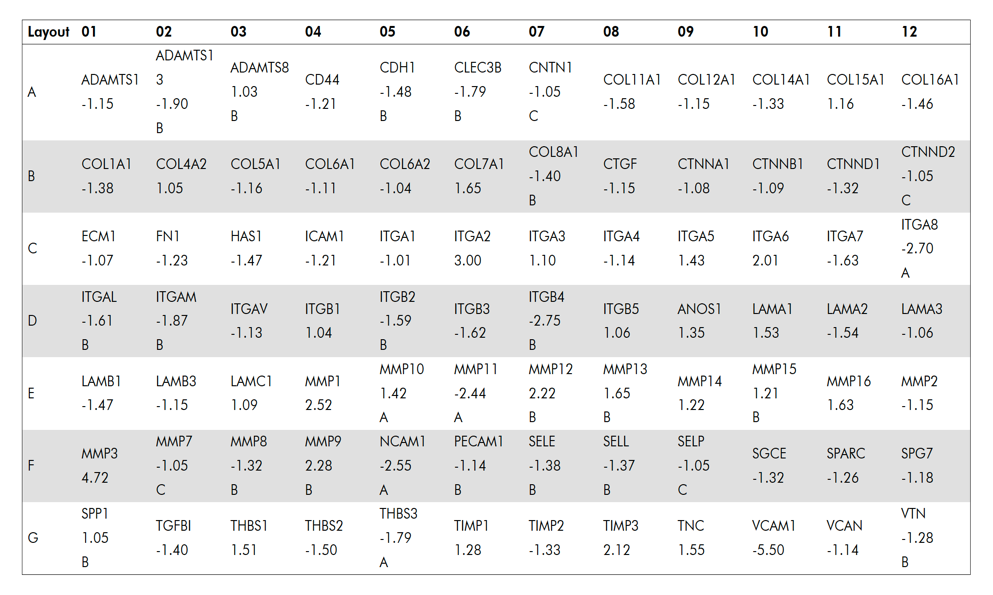**  LR-PRP versus serum-free (negative control)  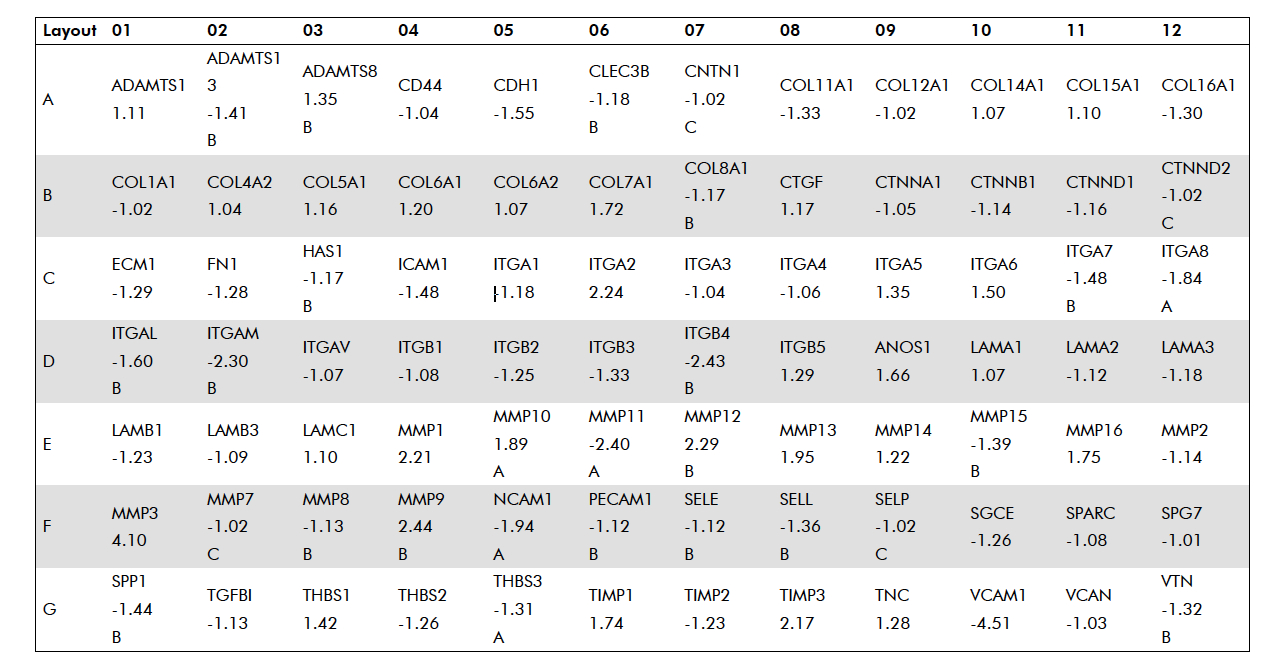 |
| --- |

**Figure S1.** All 84 evaluated expressions of mRNA genes of human ECM and adhesion molecules are shown. Fold values more than -2 and less than 2 were deemed non-significant. The housekeeping genes panel and other genes used for normalisation of the raw data are not presented. Letter A specifies that the gene’s average threshold cycle is relatively high (> 30) in either the control or the test sample and is reasonably low in the other sample (< 30). These data mean that the gene’s expression is relatively low in one sample and reasonably detected in the other sample, which suggests that the actual fold-change value is at least as large as the calculated and reported fold-change result. Letter B specifies that the gene’s average threshold cycle is relatively high (> 30), meaning that its relative expression level is low, in both control and test samples, and the p value for the fold-change is either unavailable or relatively high (p > 0.05). Letter C specifies that the gene’s average threshold cycle is either not determined or greater than the defined cut-off (35) in both samples, meaning that its expression was undetected, making this fold-change result erroneous and un-interpretable.
